# T Lymphocyte Serotonin 5-HT_7_ Receptor Is Dysregulated in Natalizumab-Treated Multiple Sclerosis Patients

**DOI:** 10.3390/biomedicines10102418

**Published:** 2022-09-27

**Authors:** Flora Reverchon, Colleen Guillard, Lucile Mollet, Pascal Auzou, David Gosset, Fahima Madouri, Antoine Valéry, Arnaud Menuet, Canan Ozsancak, Maud Pallix-Guyot, Séverine Morisset-Lopez

**Affiliations:** 1 UMR7355, Experimental and Molecular Immunology and Neurogenetics, CNRS and University of Orléans, 45071 Orleans, France; 2 UPR4301, Center for Molecular Biophysics, CNRS, 45071 Orleans, France; 3 Neurology Department, Regional Hospital Orleans, 45100 Orleans, France; 4 Medical Information Department, Regional Hospital Orleans, 45100 Orleans, France

**Keywords:** serotonin, 5-HT_7_ receptor, multiple sclerosis, natalizumab treatment, T lymphocyte (T-cell), PBMCs (peripheral blood mononuclear cells), interleukin-10 (IL-10)

## Abstract

Serotonin (5-HT) is known as a potent immune cell modulator in autoimmune diseases and should be protective in the pathogenesis of multiple sclerosis (MS). Nevertheless, there is limited knowledge about receptors involved in 5-HT effects as well as induced mechanisms. Among 5-HT receptors, the 5-HT_7_ receptor is able to activate naïve T cells and influence the inflammatory response; however, its involvement in the disease has never been studied so far. In this study, we collected blood sample from three groups: acute relapsing MS patients (ARMS), natalizumab-treated MS patients (NTZ), and control subjects. We investigated the 5-HT_7_ expression on circulating lymphocytes and evaluated the effects of its activation on cytokine production with peripheral blood mononuclear cell (PBMC) cultures. We found a significant increase in the 5-HT_7_ surface expression on T lymphocytes and on the different CD4^+^ T cell subsets exclusively in NTZ-treated patients. We also showed that the selective agonist 5-carboxamidotryptamine (5-CT)-induced 5-HT_7_R activation significantly promotes the production of IL-10, a potent immunosuppressive cytokine in PBMCs. This study provides for the first time a dysregulation of 5-HT_7_ expression in NTZ-MS patients and its ability to promote IL-10 release, suggesting its protective role. These findings strengthen the evidence that 5-HT_7_ may play a role in the immuno-protective mechanisms of NTZ in MS disease and could be considered as an interesting therapeutic target in MS.

## 1. Introduction

The neurotransmitter serotonin (5-HT) acts primarily by binding to its G-protein-coupled receptors divided into seven subfamilies (5-HT_1_ to 5-HT_7_, except the 5-HT_3_, which is a ligand-gated ion channel) whose expression extends to several organs such as the lungs, intestine, liver, and both the peripheral and central nervous systems (CNS). This broad serotonin system representation allows 5-HT to influence many physiological functions with specific effects to the activated 5-HT receptor and the targeted cell type [1]. Moreover, 5-HT is able to modulate the immune response, both innate and adaptive, through its receptors expressed on the surface of immune cells, including neutrophils, eosinophils, monocytes, macrophages, dendritic cells, mast cells, and natural killer cells [2]. The chemoattractant properties of 5-HT are mediated by 5-HTR_1A_ and 5-HTR_2A_ pathways on human mast cells and eosinophils, respectively. Moreover, it has been demonstrated that 5-HT depletion using parachlorophenylalanine limits the priming adaptative immune cells by blocking T cell activation by macrophages [2].

Thus, 5-HT was shown to play an immune-modulatory role in the pathogenesis of autoimmune diseases such as multiple sclerosis (MS) [3], without understanding precisely the molecular events involved.

MS is a chronic, immune-mediated, inflammatory, and demyelinating disease of the CNS, with immune cell reactivity against the myelin sheath of neuronal axons. The invasion of lymphocytes is correlated with cytokine activity in the CNS, with disease activity associated to higher expression of inflammatory cytokines such as IFN-γ, IL-1β, IL-17, IL-22, and TNF [4]. A major involvement of CD8^+^ and CD4^+^ T infiltrated cells has been demonstrated in the MS pathogenesis and inflammatory relapses, associated with deleterious pro-inflammatory roles of CD4^+^ T_H_1 and CD4^+^ T_H_17 T cell subpopulations [4]. This immunological imbalance seems to be modified by exogenous 5-HT in favor of an immunomodulatory response by regulatory CD4^+^ T cells (Treg) [5]. Much attention has been devoted to identifying the 5-HT receptors and signaling pathways involved. Recent studies demonstrated the anti-inflammatory role of 5-HT_1A_ and 5-HT_2B_ receptors on the CD4^+^ T cell surfaces in MS [6,7]. Nevertheless, the mechanisms by which 5-HT modifies autoimmune responses remain poorly understood [3,8].

Among the 5-HT receptors, the most recently discovered, the 5-HT_7_ receptor, seems to be an interesting candidate in the CNS. Indeed it has been shown to regulate important pathophysiological processes and has become a promising target in the treatment of CNS disorders such as sleep disorders, migraine, neuropathic pain, or neuro-psychiatric disorders [9,10]. Moreover, the 5-HT_7_ receptor presence on the surface of innate and adaptive immune cells [2] suggests its role in immune-mediated disorders [1]. The activation of 5-HT_7_ receptor induced by 5-HT endogenous signal has been shown to promote T-cell activation and proliferation [11], as well as the regulation of cytokine releases such as TNF-α and IFN-γ [1]. More recently, the 5-HT_7_ receptor has been shown to be expressed on the surface of accumulated Treg cells in the mouse brain after ischemic stroke. In this context, the 5-HT_7_ receptor could participate in the moderating and neuroprotective roles of Treg cells [11]. Therefore, on the basis of its therapeutic potential, modulation of 5-HT_7_ activity has been the focus of numerous drug discovery programs, and several 5-HT_7_-specific ligands have been successfully developed during the past two decades [12]. Among them, the LP-44, a specific 5-HT_7_ agonist, was shown to modulate the inflammatory response by inhibiting the inflammatory cytokine releases such as TNF-α, whereas the antagonist SB-269970 reduced it [1].

Despite increasing data reporting 5-HT_7_ receptor involvement in T-cell activation and inflammation, its actual expression level in different inflammatory pathological conditions has not been extensively reported. Taking into account that 5-HT is able to modulate T-cell behavior in MS [5], our study focused on the 5-HT_7_ expression and its functions in three groups: acute relapse MS patients (ARMS) in the course of an acute inflammation, natalizumab-treated MS patients (NTZ) with a stable clinical state, and healthy subjects. We investigated the 5-HT_7_ receptor expression on the main subsets of peripheral lymphocyte by flow cytometry. We were interested in the possible correlation between the immune response in the MS context (with or without NTZ treatment) and the 5-HT_7_ receptor expression and/or function. For this purpose, we analyzed the *Htr7* gene activity in peripheral blood mononuclear cells (PBMCs) in the three groups. Then, 5-HT_7_ receptor was activated in PBMCs cultures, and we performed immunoassays to access its possible effects on immune function.

## 2. Materials and Methods

### 2.1. Standard Protocol Approvals and Subject Characterization

**Figure 1:**
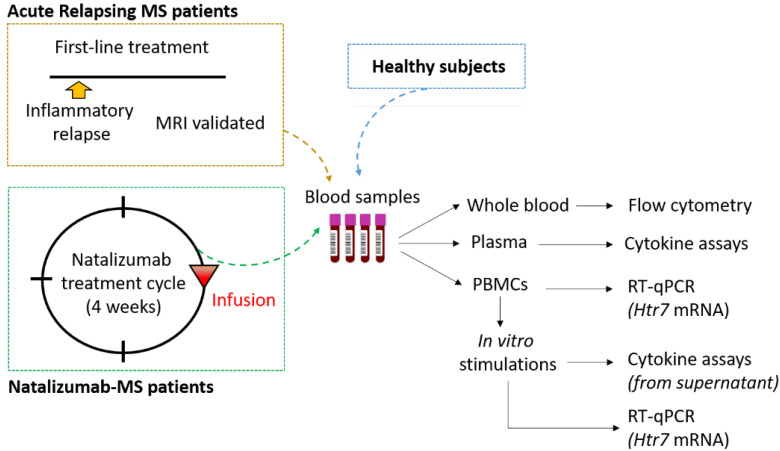
Experimental design and analyses performed. Abbreviations: PBMCs: peripheral blood mononuclear cells; RT: reverse transcription; qPCR: quantitative polymerase chain reaction; mRNA: messenger ribonucleic acid; *Htr7*: serotonin 5-HT_7_ receptor gene.

This study was carried out in accordance with the protocol validated by the Ethics Committee EST IV from Strasbourg in France. Trial registration at clinicaltrials.gov is NCT04546698. All written informed consent forms followed the ethical guidelines that the 1964 Declaration of Helsinki had set. MS patients were diagnosed according to the 2018 revised McDonald criteria [13] and were recruited from the neurology unit of the “Centre Hospitalier Régional d’Orléans”. Healthy subjects were recruited from the “Établissement Français du Sang” (Orléans, France). Starting with blood samples, the study design is summarized in Figure 1. Samples from MS patients with acute relapse were obtained during an inflammatory phase before any treatment to limit relapse. Natalizumab-treated MS patients were cured for at least 6 months, and blood samples were obtained at the end of one cycle of natalizumab (4-week cycle) and before the next infusion. They were clinically stable with normal MRI scans and a minimum of 6 months of treatment. The following demographic and clinical characteristics were evaluated in every case: age, sex, disease duration, MS group (ARMS: acute relapsing MS, NTZ: natalizumab-treated MS), disability scores (EDSS: Expanded Disability Status Scale), and treatment with selective serotonin recapture inhibitors. Healthy controls were matched for age and sex, as displayed in Table 1.

### 2.2. Analysis of 5-HT_7_ Receptor Expression on Lymphocyte Surface

5-HT_7_ receptor expression level was evaluated from whole blood using flow cytometry. We performed three tubes per patient corresponding to unlabeled blood cells, Fluorescence Minus One (FMO) controls, and cells labeled for the 5-HT_7_ receptor. All staining reactions were performed at room temperature for 20 min in the dark. Briefly, cells were first stained with specific primary antibody (Ab) for 5-HT_7_ receptor (13830-1-AP, Proteintech, Manchester, Europe). This first step concerns only labeled cells. The rest of the protocol concerns labeled cells and the FMO control. Then, cells were washed with Stain Buffer (554657, BD Biosciences, Paris, France), followed by centrifugation at 1500 rpm for 5 min. Secondary Ab specific to the primary (ab150077, abcam, Cambridge, England) was incubated. Leucocytes were then washed and stained using a combination of the following fluorochrome-conjugated Ab (BD Biosciences^®^), diluted in Brilliant Stain Buffer (563794): CD45-BV510 (563204, 1/200), CD19-BV786 (563325, 1/200), CD3-APC-H7 (560171, 1/200), CD8-PerCp-Cy5 (565310, 1/200), CD4-APC (566915, 1/200), CXCR3-BV711 (563156, 1/200), CCR4-BV421 (562579, 1/200), CCR6-PE (551773, 1/50), CD25-PE-CF594 (562403, 1/200), CD127-PE-Cy7 (560822, 1/200). Red blood cells were then lysed (349202, BD Biosciences) at room temperature for 10 min in the dark. Finally, cells were fixed (339860, BD Biosciences) at 4 °C for >30 min in the dark, washed, and re-suspended in Stain Buffer. Flow cytometry analyses were performed on LSR Fortessa X-20 flow cytometer (Becton Dickinson). Gating strategy was set up according to FMO control for all antibodies. One hundred thousand CD4^+^ cells were recorded in order to have a sufficient regulatory T (Treg) cell number, representing the minority subpopulation. Final analysis and graphical output were performed using FlowJo software version 10 (Tree Star, Ashland, OR, USA).

### 2.3. Isolation and Stimulation of Peripheral Blood Mononuclear Cells

Peripheral blood mononuclear cells (PBMCs) from 30 healthy controls, 30 natalizumab-treated MS patients, and 18 acute relapsing MS patients were isolated from blood samples collected in EDTA tubes using Ficoll density gradient centrifugation (11753219, Fisher Scientific, Massachusetts, United States) at 2000 rpm for 20 min. PBMCs were then quickly re-suspended in 1X PBS and washed twice with centrifugations at 1400 rpm for 7 min.

PBMCs were incubated in RPMI 1640 (GIBCO, Life Technologies, Pleasanton, CA, USA) supplemented with 1% L-glutamine, 1% pyruvate sodium, 1% penicillin–streptomycin, 1% MEM non-essential amino acids solution, and 10% heat-inactivated fetal calf serum (FCS) in 24-wellsplate at 37 °C overnight. PBMCs (2 × 10^6^ cells/well) were then stimulated with lipopolysaccharides (LPS, 100 ng/mL)-(L2630, Sigma-Aldrich, Saint-Quentin Fallavier, France) or 5-carboxamidotryptamine (5-CT, 10 µM)-(0458, TOCRIS) for 4 h at 37 °C. PBMCs and supernatants were collected at the end of stimulation and stored at −80 °C for RNA extractions and cytokine assays.

### 2.4. Cytokine Assays in Plasma Samples and PBMC Supernatants

Plasma was collected from patient’s whole blood after centrifugation at room temperature for 5 min at 1500 rpm and stored at −80 °C until the assays. ELISA (R&D systems) quantified human IL-17 (DY317-05), IL-1β (DY201-05), IFN-γ (DY285B-05), IL-10 (DY217B-05), and IL-4 (DY204-05) assays in plasmas and PBMCs supernatants, according to the manufacturer’s instructions.

### 2.5. Analysis of 5-HT_7_ Receptor Transcription Level

RNA was collected and extracted using a miRNeasy Mini Kit according to the manufacturer’s instructions (Qiagen, Hilden, Germany) from PBMCs. RNA integrity and quality were controlled with Agilent RNA 6000 Nano Chips kit^®^ (5067-1511, Agilent, Santa Clara, CA, USA). Reverse transcription was performed with SYBR^®^ Premix Ex Taq^TM^ (Takara, Kusatsu, Japan), and cDNA was subjected to quantitative real-time PCR using primers for *Htr7* (*#*QT00012481, Qiagen) and ONEGreen^®^ Fast qPCR Premix (Ozyme, Saint Cyr l’Ecole, France). Relative RNA expression was normalized to *Hprt1* and *Gapdh* expressions (Qiagen), and raw data were analyzed by the 2^∆∆Ct^ method [14].

### 2.6. Statistical Analysis

All graphic data are presented as mean ± SEM. Statistical analyses were performed with R software using, according to data distribution, either one factor within subject ANOVA with Tukey contrasts for post hoc comparisons or the Kruskal–Wallis test and pairwise Wilcoxon test with Holm–Sidak *p*-value adjustment. Statistical differences were considered significant when *p* < 0.05, with asterisks denoting the degree of significance (* *p* < 0.05; ** *p* < 0.01; *** *p* < 0.001). The graphical representations were performed with GraphPad Prism 9.0 software.

## 3. Results

### 3.1. High Surface Expression of 5-HT_7_ Receptor on T Cells in Natalizumab-Treated MS Patients

The neuroprotective role of serotonin [3] is associated with its ability to modulate the inflammatory response of lymphocytes [6] involved in MS disease course [15], but the knowledge of the receptors involved is limited. Moreover, several studies reported an important activation of immune cells in peripheral blood from MS patients, including changes in surface phenotype [16].

**Figure 2:**
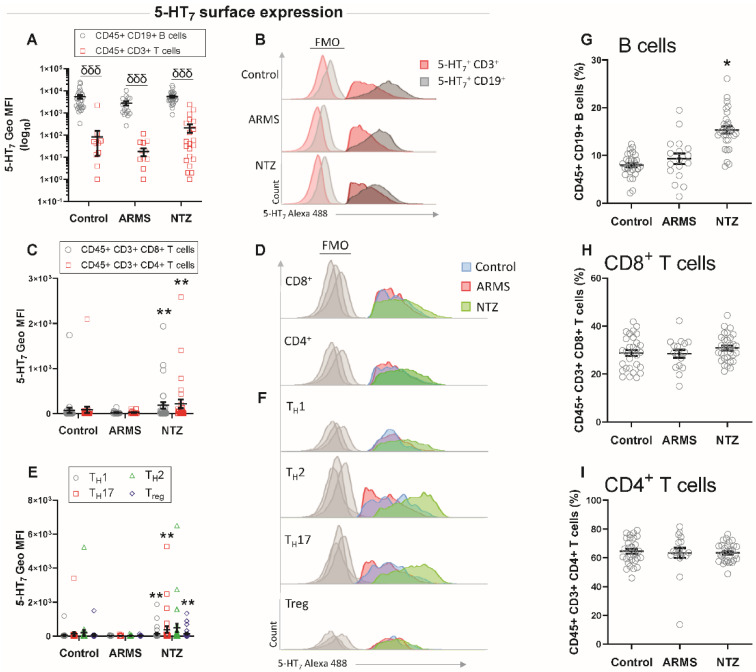
5-HT_7_ expression level on the surface of circulating lymphocytes from control, ARMS, and NTZ patients. Geometric mean fluorescence intensity (Geo MFI) of 5-HT_7_ surface expression and histogram representation of the cytometry analysis on CD45^+^ CD19^+^ B and CD45^+^ CD3^+^ T lymphocytes (**A**,**B**), on CD3^+^ CD8^+^ and CD3^+^ CD4^+^ T cells (**C**,**D**), and CD4^+^ T cell subsets (**E**,**F**). Proportions of circulating cells analyzed by flow cytometry are shown for B cells (**G**), CD8^+^ (**H**), and CD4^+^ T cells (**I**) from control, ARMS, and NTZ patients. Results are given as mean ± SEM. B cells compared with T cells for each group. *: compare with control. δδδ *p* < 0.001; * *p* < 0.05; ** *p* < 0.01.

Therefore, we investigated the surface expression levels of 5-HT_7_ on different lymphocyte populations from whole blood samples. We found that the 5-HT_7_ expression was significantly higher on the surface of CD19^+^ B lymphocytes than on CD^3+^ T lymphocytes for all three groups (*p* < 0.001, Figure 2A,B). When compared to their control counterparts, ARMS samples did not show any significant differences, while NTZ patients’ cells displayed a significant increase in the 5-HT_7_ surface expression on CD3^+^ CD8^+^ and CD3^+^ CD4^+^ T cells (*p* < 0.01, Figure 2C,D), as well as on the CD4^+^ T cell subsets CD4^+^ CD183^+^ T_H_1, CD4^+^ CD196^+^ T_H_17, CD4^+^ CD194^+^ T_H_2, and CD4^+^ CD25 (high) CD12_7_ (low) Treg (*p* < 0.01, Figure 2E,F). Next, we investigated the circulating T and B lymphocyte proportions that are supposed to be modified under natalizumab treatment, which induces compartmentalization of immune cells at the periphery [17]. We found significantly more circulating CD19^+^ B lymphocytes in the NTZ MS patients (*p* < 0.05, Figure 2G), while CD3^+^ T cells were unchanged (Figure 2H,I).

### 3.2. Decreased 5-HT_7_ Receptor Gene Activity in the Absence of a Peripheral Inflammatory Context in Natalizumab-MS Patients

The gene expression level is to be considered when its protein is modified in order to better understand the intracellular mechanisms involved. In the context of stress protocols performed on rat lymphocytes, it was observed that there was an increase in the number of 5-HT_7_-receptor-positive lymphocytes, correlated with an increased activity of the gene. The authors suggest a potential role for the receptor in stress response mechanisms involving the lymphocytes [16]. Considering our findings of increased 5-HT_7_ receptor expression on the surface of T cells in NTZ patients, we were interested in *Htr7* gene expression level within PBMCs and a possible link with the peripheral inflammatory context by measuring some plasma cytokine levels as well as in the peripheral cytokine context in the three groups.

**Figure 3:**
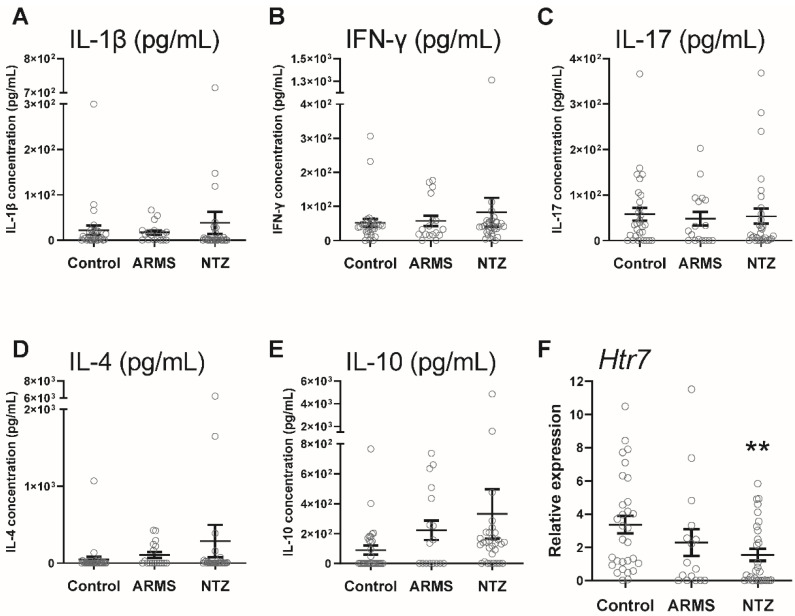
Plasma immunological context and *Htr7* gene expression in PBMCs from control, ARMS, and NTZ patients. Cytokines assays: IL-1β (**A**), IFN-γ (**B**), IL-17 (**C**), IL-4 (**D**), and IL-10 (**E**) from plasma samples of control, ARMS, and NTZ patients. *Htr7* was analyzed by RT-qPCR. The relative expression of *Htr7* mRNA levels, normalized to Gapdh and Hprt1 of control subjects, is shown (**F**). Results are given as mean ± SEM. ** *p* < 0.01 compared with control.

We assessed on one hand the peripheral inflammatory context by cytokine assays from plasma samples and on the other hand the transcriptional level of the receptor in PBMCs. We found no IL-1β, IFN-γ, IL-17, IL-4, and IL-10 level differences between the three groups (Figure 3A–E), while the 5-HT_7_ was transcribed at significantly lower levels in PBMCs of NTZ patients compared to ARMS patients and healthy subjects (*p* < 0.01, Figure 3F).

### 3.3. 5-HT_7_ Activation Promoted IL-10 Release from PBMCs under Physiological and Pathological Conditions

**Figure 4:**
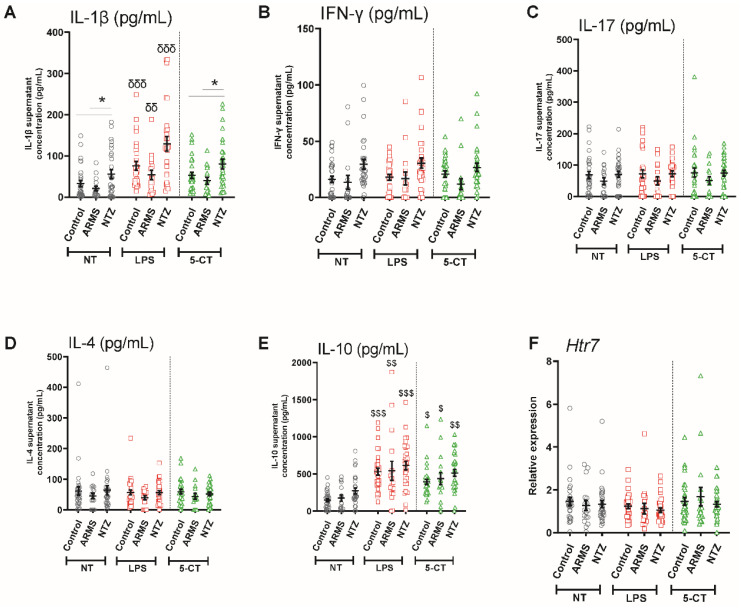
Effects of 5-HT_7_ receptor agonist 5-CT on PBMC cytokine releases compared to LPS stimulation. Cytokines assays: IL-1β (**A**), IFN-γ (**B**), IL-17 (**C**), IL-4 (**D**), and IL-10 (**E**) were performed from PBMC supernatants of control, ARMS, and NTZ patients. *Htr7* was analyzed by RT-qPCR. The relative expression of *Htr7* mRNA levels, normalized to *Gapdh* and *Hprt1* of non-treated PBMCs from the control, is shown (**F**). Results are given as mean ± SEM. *: compare the values under the bars. $: compare with the respective non-treated (NT) condition. Compare with both NT and 5-CT conditions. * *p* < 0.05; ^$^ *p* < 0.05; ^$$^ *p* < 0.01; ^$$$^ *p* < 0.001; ^δδ^ *p* < 0.01; ^δδδ^ *p* < 0.001.

To evaluate their intrinsic cytokine secretion capacity, isolated PBMCs from patients and controls were cultured outside their in vivo immunological context and maintained in vitro with an identical culture medium. Indeed, clinical and animal studies demonstrated cellular reactivity in MS, particularly that of T and B lymphocytes as well as monocytes, which is involved in the pathogenesis [18,19]. First, without any stimulation, we observed a significantly higher basal IL-1β release in the NTZ patients (*p* < 0.05), compared to the two other groups, suggesting a different activation state of NTZ PBMCs cells. Then, to determine how pathological conditions may influence the ability of PBMCs to respond to exogenous stimulation, we performed *LPS* in vitro stimulation as a positive control. As expected, we found that LPS stimulation significantly promoted the pro-inflammatory IL-1 β release in all three groups compared with the 5-CT stimulation and the untreated basal condition (*p* < 0.01, *p* < 0.001; Figure 4A). Conversely, we observed no response of LPS to induce IFN-γ, IL-17, and IL-4 productions (Figure 4B–D), and we reported a significant increase in the anti-inflammatory IL-10 release by PBMCs after LPS stimulation from all three groups compared to the basal condition (*p* < 0.01, *p* < 0.001, Figure 4E). Then, we stimulated PBMCs with the 5-HT_7_ receptor agonist 5-CT to determine the role of 5-HT_7_ receptor in PBMC cytokine release and whether these 5-HT_7_ functions could be altered in MS patients. Interestingly, 5-CT did not modify IL-1β, IFN-γ, IL-17, and IL-4 production in any group (Figure 4A–D), while it induced a specific and significant IL-10 release by PBMCs in all three groups (*p* < 0.05, *p* < 0.01; Figure 4E). Then, we evaluated the 5-HT_7_ transcriptional level under both LPS and 5-CT conditions and we did not find any significant differences for all groups (Figure 4F).

## 4. Discussion

Previous studies demonstrated that 5-HT influences the chronic deleterious inflammation in MS [20]. Supported by the MS animal model, i.e., experimental autoimmune encephalomyelitis (EAE), clinical data showed the beneficial effects of selective serotonin reuptake inhibitors on MS pathogenesis [21]. The 5-HT_7_ receptor has been described to be expressed at the surface of lymphocytes [11] and thus may be an important player of the inflammation regulation in MS mediated by lymphocytes. Nevertheless, no studies have distinguished the receptor expression on the surface of B cells versus T cells, with the two subsets having very distinct roles in the MS pathogenesis.

For the first time, we showed a higher surface expression of the 5-HT_7_ receptor on the B cells compared to the T cells for all three groups, thereby meaning this expression appears to be independent of the disease and treatment. This large B cell expression suggests that the 5-HT_7_ receptor may play a relevant physiological role on this particular lymphocyte subset. When focusing on T lymphocytes, we found that 5-HT_7_ receptor expression was significantly higher on the surface of CD4^+^ and CD8^+^ T cells in NTZ patients compared to ARMS patients and healthy subjects. Romme Christensen et al., demonstrated that immune cells in peripheral blood from progressive MS patients presented a higher level of activation with increased frequencies of ICOS^+^ T_FH_ cells and changes in surface phenotype with a larger quantity of IL-23 receptor expression on (IL23R)^+^CD4^+^ T cell surface [22]. Analysis of T_H_1, T_H_17, T_H_2, and Treg CD4^+^ T lymphocyte subpopulations confirmed the 5-HT_7_ receptor overexpression on their surface exclusively in NTZ patients. Natalizumab treatment blocks the lymphocyte infiltration toward the tissues, including CNS, resulting in changes in blood lymphocyte composition [17] with increased circulating T-cell reactivity [23,24]. Börnsen et al., provided evidence that the NTZ treatment not only alters the percentage of circulating leukocytes but may promote a direct pro-inflammatory effect on PBMCs [25]. Indeed, VLA-4 antigen binding occurring with the NTZ treatment provides a co-stimulatory signal to the cells, promoting their activation and accumulation in the periphery [26]. On the basis of these results, we show that the 5-HT_7_ receptor was overexpressed on the surface of probably reactive T cells in NTZ patients.

Interestingly, Urbina et al., showed that 5-HT_7_ overexpression on the surface of isolated rat CD4^+^ and CD8^+^ T cells after concanavalin A activation suggests a 5-HT_7_ protective role in the inflammatory context [16]. In order to understand whether the 5-HT_7_ overexpression in NTZ patients was also associated with an inflammatory context, we investigated the peripheral immunological response. We assayed five cytokines in the patient’s plasma; IL-1β, IFN-γ, and IL-17 as biomarkers of pro-inflammatory response; and IL-10 and IL-4 as anti-inflammatory response. We found no difference in the cytokine production in whole plasma samples between the three groups. A study performed on the cerebrospinal fluid (CSF) of MS patients demonstrated a systemic inflammatory response correlated with aberrant lymphocyte activation [22]. This suggests that the method we used to measure cytokine production was not sensitive enough to detect slight variations between groups. Alternatively, our results suggest that CSF is preferable to plasma for cytokine assays in neuro-inflammatory diseases such as MS [27].

The study by Urbina et al., proposed a link between 5-HT_7_ overexpression on activated isolated rat CD4^+^ and CD8^+^ T cells and a significantly increased *Htr7* gene activity [16]. In contrast, our results showed a significant decrease in *Htr7* gene activity specifically in PBMCs isolated from NTZ-treated patients compared to ARMS patients and healthy subjects. Previously, Albayrak et al., investigated the *Htr7* gene activity in carrageenan-induced inflammatory context in rats and showed that 5-HT_7_ receptor activation by agonist leads to a decrease in gene activity associated with reduction of serum TNF-α, IL-1β, and IL-6 levels [28]. These results suggested a protective mechanism through 5-HT_7_ desensitization with an *Htr7* gene activity downregulation that is common among G-protein-coupled receptors [29]. Thus, for the first time, our study showed *Htr7* gene downregulation specifically in NTZ patients whose 5-HT_7_ receptor expression on the activated T lymphocyte surfaces was much higher than that of ARMS patients and healthy subjects. Only one study has investigated desensitization by internalization of the three human 5-HT_7_ receptor isoforms and discussed the pattern of receptor trafficking in response to agonists [30]. Guthrie et al., provided evidence that the surface 5-HT_7_(d) isoform receptor are constitutively internalized in the absence of agonists, while the majority of 5-HT_7_(a) and 5-HT_7_(b) isoforms remain on the surface. Comprehensive investigations will be required to identify the 5-HT_7_-receptor-mediated intracellular pathways involved in the negative *Htr7* gene activity.

The 5-HT_7_ receptor has been shown to modulate the production of pro- and anti-inflammatory cytokines in different contexts. The receptor promotes the IL-1β release by dendritic cells, while it reduces TNF-α and IL-6 in liver chronic injury [1]. Nevertheless, no data have been provided regarding its role on the leukocyte-mediated immunological response in MS. In isolated PBMCs cultures, we first showed a significantly higher IL-1β release from NTZ patients’ PBMCs in the absence of exogenous stimulation, suggesting a basal inflammation state. Indeed, animal and clinical studies have demonstrated cellular activity in MS and particularly of B and T lymphocytes, which are involved in the pathogenesis [17,18]. In addition, it has been demonstrated that there is an immune cell proportion altered in the CSF of MS patients that is associated with inflammatory mediators such as IL-1β [31]. Then, we showed the ability of these cells to respond to in vitro positive control LPS stimulation with a massive release of both IL-1β pro-inflammatory and IL-10 anti-inflammatory cytokines to counterbalance the response.

Furthermore, we showed that the 5-HT_7_ receptor agonist 5-CT resulted in a significant increase in the anti-inflammatory IL-10 release in all three groups. Interestingly, in contrast to the LPS effect, stimulation by 5-CT did not significantly influence the IL-1β release compared to non-treated conditions, suggesting that the 5-HT_7_ receptor is not involved in this pro-inflammatory pathway mediated by IL-1β. Several recent studies provide evidence of the 5-HT_7_ receptor anti-inflammatory effect with a protective role by decreasing the pro-inflammatory cytokine release as TNF-α or by limiting cell apoptosis [1,21,22]. Moreover, it has been shown that the 5-HT_7_ receptor is associated with macrophage M2 polarization, characterized as protective cells in an inflammatory context [20]. A recent review describes the known protective roles of 5-HT_7_ receptors in neurodegenerative diseases that are associated with reduction of oxidative stress, synaptic remodeling, and immunomodulation [32]. All these reports support our results and the 5-HT_7_ receptor ability to modulate immune function in MS and beyond. Our in vitro results show that the release of IFN-γ, IL-17, or IL-4 was not induced by LPS, nor by 5-CT in all three groups, suggesting that 5-HT_7_-receptor-mediated signaling pathways are not directly involved in these cytokine releases. Moreover, in the two stimulation conditions, LPS or 5-CT had no effect on the *Htr7* gene activity for the three groups, suggesting an absence of in vitro regulation in our experimental conditions. Further experiments would be required to understand the *Htr7* gene regulation and the signaling pathways involved in PBMC cultured cells. Indeed, it seems that the incubation time of the 5-HT_7_ agonist could be an important factor in this regulation. Thus, it was shown that the 5-HT_7_ receptor agonist LP-44 does not induce any modification of *Htr7* gene activity after 2 h of stimulation; however, increases it after 4 h and conversely decreases it at 6 h [21,33].

The influence of natalizumab treatment on the 5-HT_7_ receptor requires further investigation. Indeed, NTZ patients’ samples were collected at the end of a 4-week cycle and before the next one. The dysregulation of 5-HT_7_ expression could also be associated with a rebound disease activity after termination of NTZ treatment [23]. In addition, the 5-HT_7_ level expression on the T lymphocyte populations and the *Htr7* gene activity observed during an inflammatory attack in the ARMS group were similar to those of the healthy subjects. We cannot exclude the fact that the tissue cellular infiltration contributed to masking significant results obtained on the periphery. It would be interesting to perform further analyses in this group outside of an inflammatory phase. Moreover in future studies on the understanding of 5-HT_7_ receptor in MS, its heterodimeric form with 5-HT_1A_ [34] will be an important element to consider. Indeed, as shown by our 5-HT_7_ receptor results, the 5-HT_1A_ receptor is overexpressed on the T cell surfaces in an EAE model and is also associated with an anti-inflammatory role [7]. The involvement of the receptor present on the surface of monocytes will also be a point of clarification, as in the review by Melnikov et al., including the sometimes conflicting roles of the receptor depending on the cell type [20]. In fact, the alterations of innate immunity found in the peripheral blood [22] and in the CSF [31] of MS patients are evidence that further study is needed to determine the 5HT7 receptor involvement on the surface of innate immune cells.

To conclude, this study shows for the first time that the 5-HT_7_ receptor specifically is upregulated on the surface of CD8^+^ T cells and CD4^+^ T cell subpopulations in clinical stable MS patients treated with NTZ for at least 6 months. In these patients, we observed an *Htr7* gene expression downregulation that could be linked to G-protein-coupled receptors’ protective mechanism, even though signaling pathways have not yet been elucidated. Furthermore, common to all three groups, an increase in IL-10 release was evidenced, produced by in vitro 5-CT-treated PBMCs. This suggests an anti-inflammatory effect of 5-HT_7_ receptor through IL-10 release. Moreover, IL-10 release has been shown to be elevated in serum after first NTZ infusion, associated with beneficial effects of treatment [35]. Thus, our study suggests that reactive T cells [17,18], which are peripherally blocked by NTZ treatment, overexpress the 5-HT_7_ receptor and may promote IL-10 release, which the system needs to restore immunological balance. Altogether, our findings provide the first evidence for 5-HT_7_ implication in MS patients under NTZ treatment.

## Figures and Tables

**Table 1:** Clinical characteristics of MS patients and healthy subjects.

Clinical Characteristics	Natalizumab Treated MS Patients (*n* = 30)	Acute Relapsing MS Patients (*n* = 18)	Healthy Subjects (*n* = 30)
Age, y, mean (SEM)	37.8 (1.86)	34.1 (2.13)	39.2 (1.57)
Female, *n* (%)	25 (83)	13 (81)	25 (83)
MS symptom onset, y (SEM)	8.6 (1.24)	5.4 (1.03)	/
EDSS score, mean (SEM)	2.3 (0.40)	2.8 (0.50)	/
SSRI treatment, *n* (%)	7 (23)	1 (6)	/
Natalizumab infusions, *n* (SEM)	49 (7.55)	/	/

Abbreviations: MS: multiple sclerosis; EDSS: Expanded Disability Status Scale; SSRI: selective serotonin recapture inhibitor; /: not applicable.

## Data Availability

Data is contained within the article.
